# E-HEalth treatment in Long-term Dialysis (E-HELD): study protocol for a multicenter randomized controlled trial evaluating personalized Internet-based cognitive-behavioral therapy in dialysis patients

**DOI:** 10.1186/s13063-022-06392-9

**Published:** 2022-06-07

**Authors:** Judith Tommel, Andrea W. M. Evers, Henk W. van Hamersvelt, Sandra van Dijk, Niels H. Chavannes, Lieke Wirken, Luuk B. Hilbrands, Henriët van Middendorp

**Affiliations:** 1 Health, Medical and Neuropsychology Unit, Institute of Psychology, Faculty of Social and Behavioural Sciences, Leiden University, Wassenaarseweg 52, 2333 AK Leiden, the Netherlands; 2 Department of Nephrology, Radboud Institute for Health Sciences, Radboud university medical center, Geert Grooteplein Zuid 10, 6525 GA Nijmegen, the Netherlands; 3 Public Health and Primary Care, Leiden University Medical Center, Albinusdreef 2, 2333 ZA Leiden, the Netherlands

**Keywords:** Kidney failure, Dialysis, Internet-based cognitive-behavioral therapy (ICBT), Screening, Patient-centered care, Personalized medicine, Randomized controlled trial (RCT)

## Abstract

**Background:**

Kidney failure and dialysis treatment have a large impact on a patient’s life. Patients experience numerous, complex symptoms and usually have multiple comorbid conditions. Despite the multitude of problems, patients often have priorities for improvement of specific aspects of their functioning, which would be helpful for clinicians to become informed of. This highlights a clear need for patient-centered care in this particular patient group, with routine screening as a vital element to timely recognize symptoms and tailored treatment to match individual patients’ needs and priorities. By also providing feedback on patient’s screening results to the patient itself, the patient is empowered to actively take control in one’s mostly uncontrollable disease process. The current paper describes the study design of a multicenter randomized controlled trial evaluating the effectiveness of the “E-HEealth treatment in Long-term Dialysis” (E-HELD) intervention. This therapist-guided Internet-based cognitive-behavioral therapy (ICBT) intervention is focused on and personalized to the myriad of problems that dialysis patients experience and prioritize.

**Methods:**

After a screening procedure on adjustment problems, 130 eligible dialysis patients will be randomized to care as usual or the E-HELD intervention. Patients will complete questionnaires on distress (primary outcome measure), several domains of functioning (e.g., physical, psychological, social), potential predictors and mediators of treatment success, and the cost-effectiveness of the intervention, at baseline, 6-month follow-up, and 12-month follow-up. In addition, to take account of the personalized character of the intervention, the Personalized Priority and Progress Questionnaire (PPPQ) will be administered which is a personalized instrument to identify, prioritize, and monitor individual problems over time.

**Discussion:**

The present study design will provide insight in the effectiveness of tailored ICBT in patients with kidney failure who are treated with dialysis. When proven effective, the screening procedure and the subsequent ICBT intervention could be implemented in routine care to detect, support, and treat patients struggling with adjustment problems.

**Trial registration:**

NL63422.058.17 [Registry ID: METC-LDD]

NL7160 [Netherlands Trial Register; registered on 16 July 2018]

## Administrative information

Note: the numbers in curly brackets in this protocol refer to SPIRIT checklist item numbers. The order of the items has been modified to group similar items (see http://www.equator-network.org/reporting-guidelines/spirit-2013-statement-defining-standard-protocol-items-for-clinical-trials/).

**Table:** 

Title {1}	E-HEalth treatment in Long-term Dialysis (E-HELD): study protocol for a multicenter randomized controlled trial evaluating Internet-based cognitive-behavioral therapy in dialysis patients
Trial registration {2a and 2b}	Netherlands Trial Register (NL7160). Registered on July 16, 2018.
Protocol version {3}	Version 7.0. Date: January 29, 2020.
Funding {4}	This work was supported by the Dutch Kidney Foundation (SWO 16.07).
Author details {5a}c	**Judith Tommel**, Health, Medical and Neuropsychology unit, Institute of Psychology, Faculty of Social and Behavioural Sciences, Leiden University, Leiden, the Netherlands. **Andrea W. M. Evers**, Health, Medical and Neuropsychology unit, Institute of Psychology, Faculty of Social and Behavioural Sciences, Leiden University, Leiden, the Netherlands. **Henk W. van Hamersvelt**, Department of Nephrology, Radboud Institute for Health Sciences, Radboud university medical center, Nijmegen, the Netherlands. **Sandra van Dijk**, Health, Medical and Neuropsychology unit, Institute of Psychology, Faculty of Social and Behavioural Sciences, Leiden University, Leiden, the Netherlands. **Niels H. Chavannes**, Public Health and Primary Care, Leiden University Medical Center, Leiden, the Netherlands. **Lieke Wirken**, Health, Medical and Neuropsychology unit, Institute of Psychology, Faculty of Social and Behavioural Sciences, Leiden University, Leiden, the Netherlands. **Luuk B. Hilbrands**, Department of Nephrology, Radboud Institute for Health Sciences, Radboud university medical center, Nijmegen, the Netherlands. **Henriët van Middendorp**, Health, Medical and Neuropsychology unit, Institute of Psychology, Faculty of Social and Behavioural Sciences, Leiden University, Leiden, the Netherlands.
Name and contact information for the trial sponsor {5b}	Health, Medical and Neuropsychology unit, Institute of Psychology, Faculty of Social and Behavioural Sciences, Leiden University, Wassenaarseweg 52, 2333 AK Leiden, the Netherlands
Role of sponsor {5c}	The funder and sponsor played no role in the design of the study, the collection, management, analysis, and interpretation of data and in writing the manuscript.

## Introduction

### Background and rationale {6a}

Chronic kidney disease (CKD) is a growing, public health threat with an estimated global prevalence of 11–13% [1]. Risk factors of CKD include older age, diabetes mellitus, hypertension, cardiovascular disease, and obesity [2]. The key characteristic of CKD is a gradual loss of kidney function. When kidney function is declined to less than 15% of normal functioning, most patients become dependent on life-sustaining renal replacement therapy—dialysis or kidney transplantation. This final stage of CKD is referred to as kidney failure and is related to high symptom burden and a low quality of life (QOL) [3–5]. Symptoms include fatigue, sleep disturbances, itch, muscle cramps, pain, worrying, depressed mood, social difficulties, and changes in sexual functioning [5–9]. Together with high comorbidity in most patients, this large amount of symptoms makes it difficult for clinicians to detect the symptoms most troubling to patients, which could lead to symptoms being underrecognized or underestimated [10]. This is particularly problematic since increased symptom burden is found to be one of the most important predictors of dialysis patients’ low QOL [11, 12].

To improve the recognition of symptoms, routine questionnaire screenings are vital to detect symptoms that would be overlooked otherwise. Additionally, routine screening would facilitate patient-clinician communication by making it easier for patients to discuss their difficulties and needs [9, 13]. To simplify screening procedures, an online screening tool could be efficient, reduces missing values, and offers the possibility to present feedback in a way that is easy to understand, for example, by visualizing patients’ results [9, 14]. Subsequently, when results indicate a need for support, clinicians can offer a tailored treatment plan to meet individual patient needs.

With regard to the dialysis population, the value of tailoring treatment to individual patient needs is supported by a previous study of our group that found a high variety of reported problems and priorities for improvement between male and female patients, different age groups, and dialysis types [15]. In addition to these differences in priorities, the dialysis population is known for its high heterogeneity and comorbidity, which necessitates a personalized approach [16]. Moreover, besides increased motivation, adherence, and patient satisfaction, tailored or personalized treatment is found to result in stronger and longer-lasting effects compared to standardized treatment [17–20].

Cognitive-behavioral therapy (CBT) is a well-established treatment for psychological problems and psychiatric disorders and is increasingly used in patients with chronic conditions [21, 22]. In patients with chronic conditions, CBT can be used to support patients in adjustment to illness, including feelings of helplessness and loss of control, coping with uncertainty about the future, and adjusting to the need of medical or emotional support [23]. Other indications for CBT involve comorbid psychiatric disorders, difficulties in adherence to treatment, and problems related to illness behaviors which entails the way people perceive and act on their physical symptoms [23, 24]. With regard to dialysis patients, CBT can be applied to support patients in adjusting to and coping with physical problems that are inherent to kidney failure (e.g., fatigue, itch, pain), the dialysis treatment, or comorbid conditions, such as diabetes or cardiovascular diseases. Promising results are found for CBT to improve dialysis patients’ adjustment to these physical problems as shown by improved functioning on the physical aspects of health-related quality of life (HRQOL) [25–27] and decreased symptoms of fatigue [28]. Considering psychological functioning, CBT is found to be effective in treating anxiety and depression and improving the mental aspects of HRQOL in dialysis patients [29–31].

Despite the benefits of CBT for patients with a chronic condition, some patients experience barriers that prevent them from participating in psychological interventions, such as time constraints, a lack of availability of services, financial problems, problems in transportation to care [32], and the remaining stigma on mental illness [33, 34]. A solution to overcome these barriers is to offer CBT online. In Internet-based CBT (ICBT), patients have the freedom to select the time and place that best suits their schedule, while following treatment at home lowers the threshold of seeking psychological support [35, 36]. Especially for dialysis patients—considering their numerous visits to the clinic, mobility problems, and high disease burden—this would make CBT more accessible, convenient, and less burdensome. In addition, for therapists, offering CBT online is time-efficient and enhances flexibility which, in turn, results in reduced costs and shorter waiting lists [36].

ICBT interventions usually consist of guided self-help formats in which patients are supported by a therapist through short messages, telephone, or videoconference [37]. ICBT is found to be as effective as face-to-face CBT in improving both mental (e.g., anxiety and depression) [38, 39] and physical functioning (e.g., disease-specific quality of life, irritable bowel syndrome symptoms, headache, pain severity, disability, and fatigue) [37–39] in several somatic conditions. Compared to self-guided interventions, interventions guided by a therapist are found to result in better outcomes [40, 41]: therapist support does not only increase adherence and prevents drop-out [35], it also allows for therapists to tailor the intervention to individual patient needs, which increases the effectiveness of (I)CBT [19, 35].

When it comes to kidney failure, no controlled, adequately powered studies have been performed to study the effectiveness of tailored ICBT in the dialysis population. Only two small, single-arm studies and one feasibility and acceptability randomized controlled trial were performed, focusing on one particular symptom (e.g., depression). The single-arm studies found significant improvements in depression [42, 43]. One of the studies also found improvements in anxiety, general distress, and mental HRQOL, but found no improvements in disability and disease burden [42]. With regard to feasibility, challenges concerning patients’ computer literacy, the acceptability of distress screenings, and perceived treatment need were reported [42, 44]. Hence, for ICBT to succeed, it was urged to be attentive of a patient’s computer literacy and to embed screening procedures early on in dialysis patients’ care pathway to get patients accustomed to screenings and psychosocial support [44]. Despite these challenges, patients who completed the intervention found ICBT to be highly acceptable, worth their time, and felt more confident that they could manage their symptoms. In addition, preliminary results indicated promising effects for the cost-effectiveness of ICBT [42].

Given the lack of adequately controlled and powered studies, more research is needed to evaluate the effectiveness of ICBT in the dialysis population. In the current study, we will evaluate effectiveness of the “E-HEealth treatment in Long-term Dialysis” (E-HELD) intervention. This therapist-guided ICBT intervention is focused on and personalized to the myriad of problems that dialysis patients experience.

### Objectives {7}

The current paper addresses the design and objectives of a multicenter randomized controlled trial that aims to evaluate the effectiveness of the E-HELD intervention—a guided ICBT intervention tailored to the individual needs of patients with kidney failure treated with dialysis. It is hypothesized that, compared to care as usual, the E-HELD intervention will result in a lower impact of the disease on daily life, with distress being the primary study outcome. To take account of the personalized character of the intervention, next to generic outcome measures, a personalized outcome measure will be included to identify, prioritize, and monitor individual problems over time and incorporates the most relevant HRQOL domains of dialysis patients. Also, socio-demographic and clinical characteristics, potential predictors and mediators of treatment success (self-efficacy, self-management, illness cognitions, personality traits, therapeutic relationship), and the cost-effectiveness of the intervention will be assessed.

### Trial design {8}

This protocol is a two-arm, parallel-group multicenter randomized controlled superiority trial (RCT), with an allocation ratio of 1:1. Participants will be randomly assigned to one of two groups: (1) the control group that will receive care as usual or (2) the intervention group that will receive 3–4 months of ICBT treatment.

## Methods: participants, interventions and outcomes

### Study setting {9}

The study will be conducted in two academic hospitals (Radboud university medical center, Nijmegen; Leiden University Medical Center, Leiden), two non-academic hospitals (VieCuri Medical Centre, Venlo; Bernhoven Hospital, Uden), and two dialysis centers (Dialysis Center Groningen, Groningen; Ravenstein Dialysis Centre, Ravenstein). All study sites are located in the Netherlands.

### Eligibility criteria {10}

#### Inclusion and exclusion criteria

**Fig. 1:**
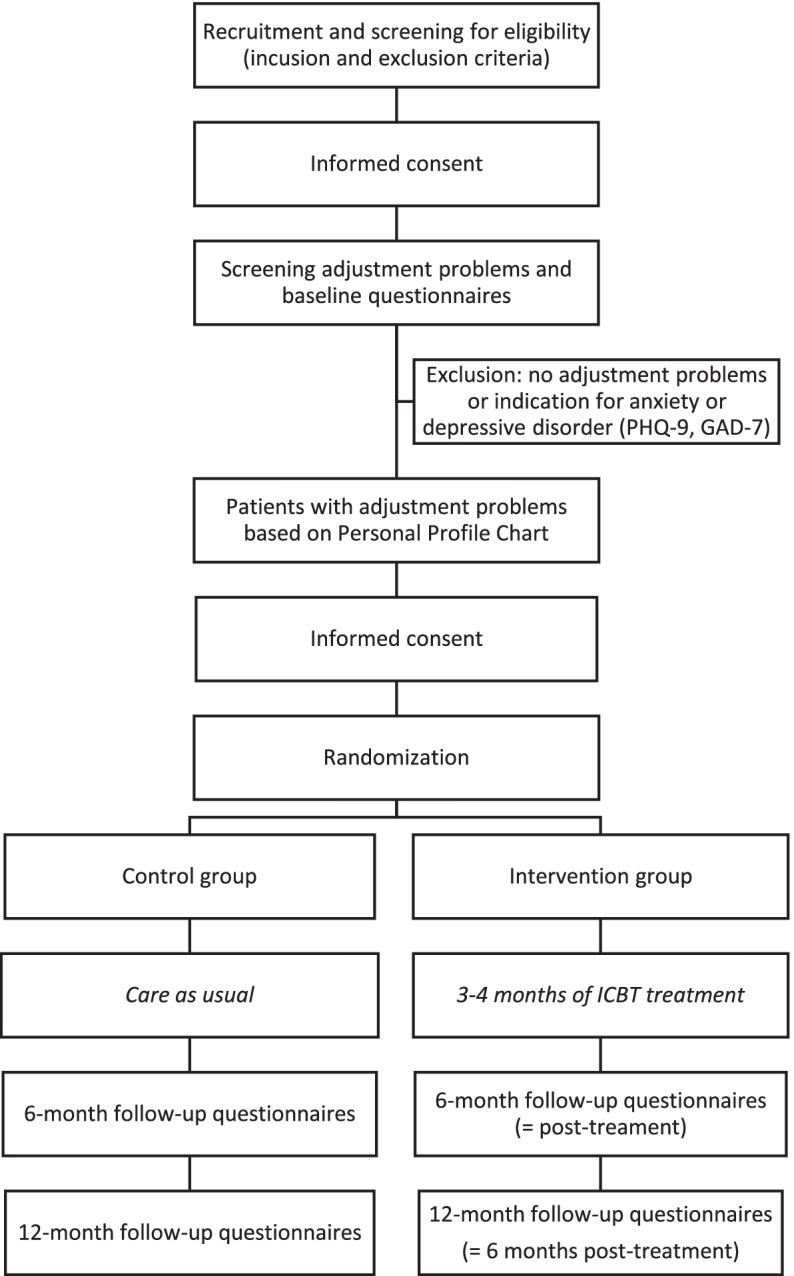
Participant timeline

See Fig. 1 for an overview of the participant timeline. The inclusion criteria are age of 18 years and older, being treated for kidney failure with hemodialysis or peritoneal dialysis for at least 3 months, and being fluent in Dutch language. Patients will be excluded when they have serious comorbid physical (life expectancy <12 months) or psychiatric conditions (i.e., DSM diagnosis), recent major stressful life events unrelated to kidney failure, and cognitive problems that would interfere with completing the questionnaires or participating in the ICBT treatment. In addition, patients will be excluded when they have a scheduled kidney transplantation within the upcoming 12 months, when they are currently receiving psychological treatment, and when they do not have access to a computer or Internet.

#### Screening of adjustment problems

Patients who meet the in- and exclusion criteria are invited to participate in the study and complete the screening and baseline questionnaires. If the screening results show adjustment problems, patients are invited to participate in the RCT. Adjustment problems are defined by physical problems, limitations in daily life, and distress. The choice for the included adjustment problems is based on a previous study on patient priorities conducted by our group [15]. See Table 1 for extended information on the study outcomes and their assessment points.

**Table 1:** Study outcomes

Domain	Measure	Explanation	Scale	Study period		
				Baseline + screening	6-month follow-up	12-month follow-up
**Primary outcome**						
Distress	Patient Health Questionnaire Anxiety and Depression Scale (PHQ-ADS) [45]	Composite measure of depression and anxiety.	0–48, higher scores indicate more symptoms of depression and anxiety, cut off: 0–9 minimal, 10–19 mild, 20–29 moderate, 30–48 severe.	X	X	X
**Secondary outcomes**						
Personalized outcome assessment	Patient Priority and Progress Questionnaire (PPPQ) [46]	Patient priorities and personal meaningful changes over time. Measured by 1 item on priorities and 8 items on functioning using 5-point Likert scales for the baseline measurement and 7-point Likert scales for the follow-up measurement. Based on the follow-up measurement, a progress score is calculated on the selected priority.	−3–3, progress score, higher scores indicate subjective improvement on the selected priority as compared to the baseline measurement.	X	X	X
Domains of functioning	RAND Short Form-36 Health Status Inventory (RAND SF-36) [47]	Health-related quality of life, including physical and mental component scores. Measured by 36 items using several Likert scales and yes/no questions.	*T*-scores, *M*=50±10 in the general population.	X	X	X
	Dialysis Symptom Index (DSI) [48]	Physical and emotional symptom burden in dialysis patients. 30 items regarding the presence of symptoms, when present symptom severity is assessed by 5-point Likert scales.	30 yes/no questions, if “yes” 1=not at all, 5=very much. Minimum score is 0 when none of the symptoms are reported. Maximum score is 150 when all 30 symptoms are reported and rated as “bothers very much” (Likert scale score 5).	X	X	X
	Subjective experience of fatigue subscale, Checklist Individual Strength (CIS) [49]	Fatigue. Measured by 8 items using 7-point Likert scales.	8–56, higher scores indicate more fatigue.	X	X	X
	Itch subscale of the Impact of Chronic Skin Disease on Daily Life (ISDL) [50]	Itch. Measured by 4 items including 3 items using 4-point Likert scales and 1 VAS (0 = no itch, 10 = worst itch ever).	4–16, the 11-point VAS will be transformed into a 4-point Likert scale to assess the total score. Higher scores indicate more itch.	X	X	X
	Inventory for Social Resilience (ISR) [51]	Social functioning. Measured by the subscales perceived support (5 items), actual support (3 items), and mutual visits (2 items) using 4-point Likert scales.	5–20, perceived support. 2–13, actual support. 2–8, mutual visits. Higher scores indicate better social functioning.	X	X	X
	Lifestyle behavior	Lifestyle behavior. Measured by 5 items using 4-point Likert scales, 2 yes/no questions, and 2 open questions to report height and weight.	7–48, total score of the 5 4-point Likert scale items and the 2 yes/no questions (2 yes/no questions are transformed to 4-point Likert scales). Higher scores indicate better lifestyle behaviors. BMI will be calculated using the patients’ height and weight, ≤18.5 underweight, 18.5–24.9 healthy weight, 25.0–29.9 overweight, ≥30.0 obese.	X	X	X
Psychological parameters	Self-Efficacy for Managing Chronic Disease 6-ltem Scale (SEMCD-6) [52]	Self-efficacy. Measured by 6 items using 10-point Likert scales.	6–60, higher scores indicate better self-efficacy.	X	X	X
	Partners in Health Scale (PiH) [53]	Chronic condition self-management knowledge and behaviors, version adjusted to chronic kidney disease. Measured by 13 items using 9-point Likert scales.	0–104, higher scores indicate better self-management.	X	X	X
	Illness Cognition Questionnaire (ICQ) [54]	Illness cognitions. Measured by the subscales helplessness (6 items), acceptance (6 items), and perceived benefits (6 items) using 4-point Likert scales.	1–24, helplessness. 1–24, acceptance. 1–24, perceived benefits. Higher scores indicate higher levels of the illness cognitions.	X	X	X
	Life Orientation Test – Revised (LOT-R) [55]	Optimism. Measured by 3 items on optimism, 3 items on pessimism, and 4 filler items using 5-point Likert scales.	0–12, 3-item optimism subscale, higher scores indicate more optimism. 0–12, 3-item pessimism subscale, higher scores indicate more pessimism. 0–24, total score 6 items, higher scores indicate more optimism.	X	X	X
	Eysenck Personality Questionnaire – Revised Short Scale (EPQ-RSS) [56], Dutch version [57]	Extraversion. Measured by 12 yes/no questions. Neuroticism. Measured by 12 yes/no questions.	0–12, extraversion, higher scores indicate more extraversion. 0–12, neuroticism, higher scores indicate more neuroticism.	X	X	X
	Internet-specific Therapeutic Relationship Questionnaire (ITRQ) [58]	Therapeutic relationship. Measured by 8 items using 10-point Likert scales.	8–80, higher scores indicate a better therapeutic relationship.		X *Intervention group only*	
Cost-effectiveness	Five-level EQ-5D (EQ-5D-5L) [59]	Health-related quality of life for economic evaluations. Measured by 5 items using 5-point Likert scales and 1 visual analog scale indicating an overall health score.	5-point Likert scales, 1=no problems, 5=unable to/extreme problems. Visual analog scale, 0=worst health you can imagine, 100=the best health you can imagine. Dutch tariff [60].	X	X	X
	iMTA Productivity Cost Questionnaire (iPCQ) [61]	Productivity losses. Measured by 12 items using open and yes/no questions and a 10-point Likert scale.	NA	X	X	X
	iMTA Medical Consumption Questionnaire (iMCQ) [62]	Medical costs. Measured by 31 items using open and yes/no questions. Depending on the answers, not all items will be presented.	NA	X	X	X
	ICEpop CAPability measure for Adults (ICECAP-A) [63]	Capability wellbeing. Measured by 5 items using 4-point Likert scales.	5–20, higher scores indicate full capability.	X	X	X
Use of ICBT intervention	Contact with e-Coach	Content, frequency, and duration of contact with the e-coach	Log data e-Coach		X *Intervention group only*	
	Evaluation of the intervention	Evaluation of the intervention. Measured by 23 items using several Likert scales and open questions.	NA		X *Intervention group only*	

##### Physical problems

Physical problems include pain, fatigue, and itch. Pain is measured by the subscale pain of the RAND Short Form-36 Health Status Inventory (RAND SF-36) [47]. Fatigue is measured by the Shortened Fatigue Questionnaire (SFQ) [49], which is a 4-item shortened version of the Checklist Individual Strength (CIS) [49]. Itch is measured by the Impact of Chronic Skin Disease on Daily Life (ISDL) [50].

##### Limitations in daily life

Limitations in daily life include limitations in daily and social activities. Both are measured by subscales of the RAND SF-36 [47]—limitations in daily activities by the subscale role limitations due to physical problems and limitations in social activities by the subscale social functioning.

##### Distress

Distress will be measured by a combination of depression and anxiety using the Patient Health Questionnaire Anxiety and Depression Scale (PHQ-ADS) [45]. This 16-item questionnaire combines the Patient Health Questionnaire depression scale (PHQ-9) [64] and the Generalized Anxiety Disorder 7-item Scale (GAD-7) [65] as a composite measure of depression and anxiety. Using this composite score, distress can be measured with one single outcome measure without needing to specify between anxiety and depression. Higher scores indicate higher levels of distress.

#### Personal Profile Chart

**Fig. 2:**
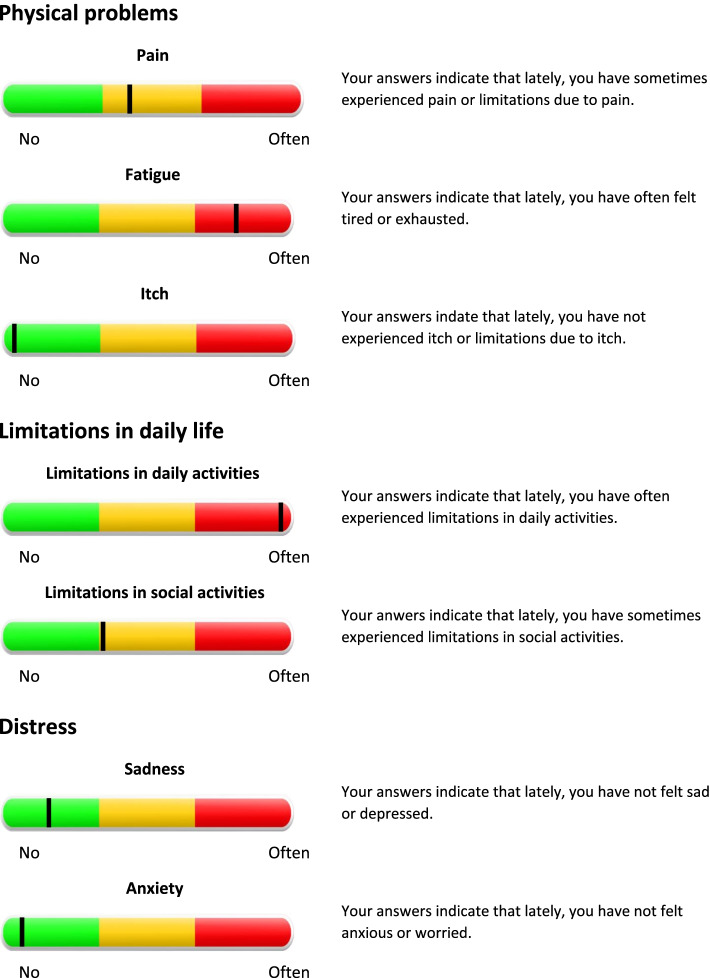
Personal Profile Chart based on screening questionnaires

Patients’ scores on the screening questionnaires are visualized in a Personal Profile Chart, using traffic light colors (i.e., red, orange, green). See Fig. 2 for an illustration of the Personal Profile Chart and Table 2 for an overview of the cut off points that were used to indicate red, orange, and green scores.

**Table 2:** Cut off points personal profile chart

Domain	Measure	Generic/disease-specific	Scale	Cut off points
Physical problems				
Pain	Pain subscale, RAND Short Form-36 Health Status Inventory (RAND SF-36) [47]	Disease-specific	20–60	Green: ≥ 47.1; Orange: 47.0–35.1; Red: ≤ 35.0
Fatigue	Shortened Fatigue Questionnaire (SFQ) [49]	Disease-specific	4–28	Green: ≤ 17,9; Orange: 18.0–23.9; Red: ≥ 24.0
Itch	Itch subscale, Impact of Chronic Skin Disease on Daily Life (ISDL) [50]	Disease-specific	3–16	Green: ≤ 5.7; Orange: 5.8–8.9; Red: ≥ 9.0
Limitation in daily life				
Limitations in daily activities	Role limitations due to physical problems subscale, RAND Short Form-36 Health Status Inventory (RAND SF-36) [47]	Disease-specific	26–56	Green: ≥ 33.1; Orange: 33.0–26.1; Red: ≤ 26.0
Limitations in social activities	Social functioning subscale, RAND Short Form-36 Health Status Inventory (RAND SF-36) [47]	Disease-specific	12–57	Green: ≥ 44.1; Orange: 44.0–31.1; Red: ≤ 31.0
Distress				
Depressed mood	PHQ-9 [64] items of the Patient Health Questionnaire Anxiety and Depression Scale (PHQ-ADS) [45].	Generic	0–27	Green: ≤ 9; Orange: 10–19; Red: ≥ 20
Anxiety	GAD-7 [65] items of the Patient Health Questionnaire Anxiety and Depression Scale (PHQ-ADS) [45]	Generic	0–21	Green: ≤ 9; Orange: 10–14; Red: ≥ 15

For the domains physical problems and limitations in daily life, percentile scores from within a similar dialysis population (*N*=172) are used to formulate cut off points [66]. Scores of the 25% of patients scoring worst on the screening measure are visualized in red and indicate frequent problems. Scores between the top 25 and 50% are visualized in orange and indicate occasional problems. Scores below the top 50 percent are visualized in green and indicates no or rare problems. For distress, existing generic cut off points for severe, moderate, and mild problems are used to determine red, orange, and green scores [64, 65]

For the domains physical problems and limitations in daily life, percentile scores from a comparable dialysis population are used to formulate cut off points. The percentile scores are based on a sample of 172 Dutch dialysis patients [66]. Scores of the 25% of patients scoring worst on the screening measure are visualized in red and indicate frequent problems. Scores between the top 25 and 50% are visualized in orange and indicate occasional problems. Scores below the top 50% are visualized in green and indicates no or rare problems.

For distress, existing generic cut off points for severe, moderate, and mild problems are used to determine red, orange, and green scores [64, 65]. Generic cut off scores were used since distress scores of this sample were comparable to that of the general population [66].

When a patient’s Personal Profile Chart shows minimally 2 orange domains and/or 1 red domain, the patient is considered eligible for participation in the RCT. Patients who have 1 or 2 red domains on emotional problems—indicating severe symptoms of depression or anxiety—will be excluded and referred to their GP. For more information on the development of the Personal Profile Chart, see the development and treatment protocol of the e-health care pathway for the CKD and dialysis population [67].

### Who will take informed consent? {26a}

During consultation or during dialysis, the nephrologist or (research) nurse will inform eligible patients about the study, answer questions, and invite patients to participate in the study. Patients will receive the patient information form, a folder about the study, and the informed consent form. Patients consenting in participation will hand in their signed informed consent form for the screening questionnaires at the hospital/dialysis center or return them by mail using the pre-stamped return envelope. After completing the screening questionnaires, patients who show adjustment problems—as indicated by the screening questionnaires—are invited to participate in the RCT. To participate in this part of the study, patients sign another informed consent form and return it by mail (see Fig. 1 for the participant timeline).

### Additional consent provisions for collection and use of participant data and biological specimens {26b}

Not applicable, this trial does not have biological specimens.

## Interventions

### Explanation for the choice of comparators {6b}

Participants will be randomly assigned to one of the two groups: (1) the control group that will receive care as usual or (2) the intervention group that will receive 3–4 months of ICBT treatment in addition to usual care. In the Netherlands, usual care for dialysis patients is based on a multidisciplinary approach by a team consisting of nephrologists, dialysis nurses, and specialized social workers that uses complaints and disease-specific PROMS (patient-reported outcome measures) as a base for guidance for medical and psychosocial problems experienced by individual patients. Specialized social workers have to work according to the Dutch Quality Standard for social workers on a dialysis unit. When deemed necessary, additional consultation by a medical psychologist or psychiatrist is routinely available in all Dutch dialysis centers. More information on the Dutch guidelines on renal replacement therapy can be found at https://www.nefrovisie.nl/richtlijnen-indicatoren/.

### Intervention description {11a}

#### Procedure E-HELD intervention

Participants allocated to the intervention condition will receive tailored ICBT. The ICBT intervention is guided by a trained psychologist, referred to as the “e-Coach.” All e-Coaches participating in this study have a master’s degree in health or clinical psychology and are supervised by a senior clinical psychologist with post-academic training in CBT. Treatment starts with a face-to-face intake meeting with the e-Coach. When a face-to-face meeting is not possible, the e-Coach will schedule a digital, videoconferencing meeting instead. During the intake, the patient will receive feedback on the screening results using the Personal Profile Chart in which the results are visualized. Based on the discussion of patient’s results, the patient and the e-Coach will determine patient’s top priorities for improvement and formulate treatment goals. Subsequently, the e-Coach will tailor the intervention so that it meets the patient’s needs. In addition, the e-Coach will explain the online environment and explores the patient’s wishes with regard to the intensity and the form of the treatment (e.g., the amount of exercises, a wish for additional phone calls, or a preference for reading texts or practical exercises). Subsequently, the patient will work on the homework assignments for 3 to 4 months.

#### Characteristics E-HELD intervention

The E-HELD intervention includes modules on coping with fatigue, itch, pain, physical disabilities, negative mood, social relations, and lifestyle. In consultation between patient and e-Coach, one or two modules will be chosen based on the screening results and patient’s priorities. Consequently, every patient’s treatment has a unique character, especially since within each module different exercises can be chosen. Exercises include several homework assignments such as reading psychoeducational texts and completing personal assignments (e.g., keeping a diary of daily activities) and exercises (e.g., relaxation). Using a message box in the online environment, the e-Coach will provide weekly personalized feedback on the exercises and send motivational messages. The patient can respond to the e-Coach’s messages or ask questions via the same message box. After 3 to 4 months, the e-Coach will contact the patient for an end-of-treatment consultation to end treatment with a final module on setting long-term goals and preventing relapse.

All modules consist of evidence-based cognitive-behavioral techniques that target risk and resilience factors and were developed from standardized CBT protocols of patients with various chronic somatic conditions [68–70]. Studies on psoriasis and rheumatoid arthritis using this intervention showed promising effects on improved physical functioning and a reduced impact of disease on daily life [70, 71]. For the current study, we tailored the intervention to the needs of dialysis patients based on the results of a previous study on patient priorities [15]. Additionally, we adjusted the itch module making it suitable for dialysis patients and added lifestyle modules to support patients in adherence to medication, physical exercise, diet restrictions, and to quit smoking. For more information on the development of the intervention for the CKD and dialysis population, please see the development and treatment protocol [67].

### Criteria for discontinuing or modifying allocated interventions {11b}

When a patient’s health significantly worsens over the course of the study, the e-Coach will discuss together with the patient to either discontinue treatment, set the treatment on hold with the possibility to continue in the future, or continue treatment in an adjusted manner (e.g., lower the amount of assignments).

### Strategies to improve adherence to interventions {11c}

We will regularly ask patients to their needs and wishes in order to optimize the feasibility and the tailored character of the treatment, making the treatment personally relevant for patients. To track adherence, the e-Coach will monitor completed assignments, modules, and the amount of chat messages.

### Relevant concomitant care permitted or prohibited during the trial {11d}

Not applicable. Usual care will be continued during the course of the trial.

### Provisions for post-trial care {30}

This study does not provide post-trial care.

### Outcomes {12}

At baseline, information on socio-demographic and clinical characteristics (age, sex, education level, employment and marital status, dialysis type and duration, comorbidity, and current or previous psychological treatment) will be collected. The Charlson Comorbidity Index [72]—a measure for comorbid conditions with weighted scores for the condition—will be calculated based on patients’ medical records. In addition, we will assess computer and Internet use and treatment expectancies regarding the ICBT treatment by means of self-report. For extended information on the study outcomes and the assessment moments, see Table 1.

#### Primary outcome: distress

The primary outcome is distress, as measured by a combination of anxiety and depression using the Patient Health Questionnaire Anxiety and Depression Scale (PHQ-ADS) [45]. The 16-item PHQ-ADS combines the PHQ-9 [64] and the GAD-7 [65] as a composite measure of depression and anxiety. Higher scores indicate higher levels of depression and anxiety symptoms. Distress will be assessed at baseline, 6 months follow-up (i.e., post-treatment for the intervention group), and 12 months follow-up (i.e., 6 months post-treatment for the intervention group).

#### Secondary outcomes

All secondary outcomes will be assessed at baseline, 6 months follow-up, and 12 months follow-up (Table 1).

#### Personalized outcome assessment

The Personalized Priority and Progress Questionnaire (PPPQ) [46] is a personalized instrument to identify, prioritize, and monitor individual problems over time. The baseline measurement consists of 8 items on several domains of functioning that have proven relevant for dialysis patients in a previous study in the Netherlands, including physical health (e.g., fatigue, pain, itch), mental health (e.g., anxiety, depression), social functioning (e.g., dependence on others), and daily activities (e.g., work, hobbies) [15]. In addition, the PPPQ includes 1 item on priorities: patients are asked to select the domains they prioritize for improvement by making a top 3. The follow-up measurement assesses the amount of progress in the areas of functioning over the past 6 months (8 items). Additionally, patients indicate on which of these areas they actively worked in the previous months (1 item). Higher scores on the follow-up measurement indicate improved functioning.

#### Domains of functioning

##### Health-related quality of life

The RAND Short Form-36 Health Status Inventory (RAND SF-36) [47] will be used to measure health-related quality of life (HRQOL). The RAND SF-36 contains 36 items that can be summarized into two summary scales: the physical component score (PCS) and the mental component score (MCS). PCS consists of the subscales physical functioning, role limitations due to physical problems, pain, and general health. MCS includes the subscales emotional wellbeing, role limitations due to emotional problems, social functioning, and vitality. Higher scores indicate a better HRQOL. The Hays norm-based scoring algorithm will be applied to transform raw scores into *T*-scores (M = 50 ± 10 in the general population) [47].

##### Symptom burden

The Dialysis Symptom Index (DSI) [48] assesses physical and emotional symptom burden in patients receiving dialysis. The DSI includes 30 items on the presence of symptoms. When present, symptom severity is rated on a 5-point Likert scale. Higher scores indicate higher symptom severity.

##### Fatigue

The subjctive experience of fatigue subscale of the Checklist Individual Strength (CIS) [49] will be used to assess fatigue. This subscale contains 8 items, with higher scores indicating more fatigue.

##### Itch

The itch subscale of the Impact of Chronic Skin Disease on Daily Life (ISDL) [50] will be used to assess itch. This subscale contains 4 items including an 11-point VAS (0 = no itch, 10 = worst itch ever). Higher scores indicate more itch.

##### Social functioning

Social functioning will be assessed by the subscales perceived support (5 items), actual support (3 items), and mutual visits (2 items) of the Inventory for Social Resilience [51]. Higher scores indicate better social functioning.

##### Lifestyle behavior

Relevant lifestyle behaviors for dialysis patients will be assessed using 8 self-constructed items. Items on diet, fluid restrictions, medication adherence, and exercise are answered on a 4-point Likert scale (1 = always, 4 = never). An example item is “I take my medication as prescribed.” Smoking behavior and weight are examined by yes/no questions (“I smoke,” “I have a healthy weight”). In addition, patients note their height and weight for BMI calculations.

### Other outcomes

All other outcomes will be assessed at baseline, 6 months follow-up, and 12 months follow-up (Table 1).

#### Psychological parameters

##### Self-efficacy

Self-efficacy will be measured by the Self-Efficacy for Managing Chronic Disease 6-ltem Scale (SEMCD-6) [52]. Higher scores indicate better self-efficacy.

##### Self-management

The Partners in Health Scale (PiH) [53] assesses chronic condition self-management knowledge and behaviors. The version of the PiH used in the study is adjusted to CKD and consists of 13 items. In this version, the illness is referred to as “kidney disease” and includes an additional item on dealing with the emotional consequences of having a kidney disease. Higher scores indicate better self-management.

##### Illness cognitions

Illness cognitions are measured by the Illness Cognition Questionnaire (ICQ) [54]. Illness cognitions reflect ways of reevaluating the aversive character of a chronic disease. The 18-item ICQ includes three generic illness cognitions: helplessness (emphasizing the aversive meaning of the disease), acceptance (diminishing the aversive meaning by learning how to cope with the disease), and perceived benefits (adding positive meaning to the disease, e.g., personal growth). Higher scores reflect higher levels of the illness cognitions.

##### Optimism

The Life Orientation Test – Revised (LOT-R) [55] is used to assess optimism. The LOT-R includes 10 items. Higher scores indicate more optimism.

##### Extraversion and neuroticism

The subscales extraversion and neuroticism of the Dutch version [57] of the Eysenck Personality Questionnaire – Revised Short Scale (EPQ-RSS) [56] will be used to measure both personality traits. Both subscales include 12 items, with higher scores indicating more extraversion and neuroticism.

##### Therapeutic relationship

The Internet-specific Therapeutic Relationship Questionnaire (ITRQ) [58] measures the Internet-specific aspects of the therapeutic relationship during Internet-based interventions. The ITRQ includes two subscales: Internet-specific time and attention (i.e., time lag aspects in communication and the sufficiency of the therapist’s attention), and Internet-specific reflection and comfort (i.e., sharing information with the therapist and the treatment environment being a patient’s home). The ITRQ includes 8 items, with higher scores indicating a better therapeutic relationship. In contrast to the other measures, the ITRQ will be completed only by patients allocated to the intervention condition. Assessment will take place at post-treatment.

#### Cost-effectiveness

##### Health-related quality of life for economic evaluations

The Five-level EQ-5D (EQ-5D-5L) [59] measures HRQOL on five dimensions of health: mobility, self-care, usual activities, pain/discomfort, and anxiety/depression. Scores of this 5-item questionnaire can be translated into quality-adjusted life years (QALYs) for health technology assessments and economic evaluations [60].

##### Productivity losses and medical costs

Two questionnaires of the Institute for Medical Technology Assessment (iMTA) will be used for the measurement of costs in economic evaluations: the iMTA Productivity Cost Questionnaire (iPCQ) [61] and the iMTA Medical Consumption Questionnaire (iMCQ) [62]. The iPCQ assesses productivity losses and includes 12 items which will be presented based on the patient’s answers. The iMCQ is a generic instrument for measuring medical costs. The iMCQ includes questions on the type and frequency of treatment, contact with health care providers, hospitalization, and medication use. The iMCQ includes 31 items—items will be presented based on the answers on the previous items.

##### Capability wellbeing

The ICEpop CAPability measure for Adults (ICECAP-A) [63] is a measure for capability wellbeing for the general adult population for use in economic evaluations. It includes 5 items on stability, attachment, autonomy, achievement, and enjoyment.

### Participant timeline {13}

See Fig. 1 for the SPIRIT figure with the participant timeline.

### Sample size {14}

The power calculation is based on the difference in change of distress from baseline to follow-up between the intervention and the control group. The effect size was based on previous RCTs on (Internet-based) CBT treatment in dialysis patients with a focus on distress-related outcomes (e.g., depression) indicating medium effects [73, 74]. In the absence of studies focusing on a broader spectrum of adjustment problems such as the current study, we used these studies for an estimation of the effect size despite the fact that they focused solely on distress. When using an alpha level of .05 and an effect size of 0.50 (Cohen’s *d* moderate effect), and taking account of potential dropouts, effects for 2 groups of 65 patients yield a power of at least .80.

### Recruitment {15}

Patient recruitment will take place in several centers in the Netherlands: Radboud university medical center, Nijmegen; Leiden University Medical Center, Leiden; VieCuri Medical Centre, Venlo; Bernhoven Hospital, Uden; Dialysis Center Groningen, Groningen; and Ravenstein Dialysis Centre, Ravenstein. Patients will be recruited from February 2019 until November 2021. Depending on the rate of inclusion, we will contact other centers as well. After screening on the inclusion and exclusion criteria, nephrologists or (research) nurses recruit eligible patients during consultation or dialysis treatment. After 3–6 months, screening will be repeated to include newly started dialysis patients.

## Assignment of interventions: allocation

### Sequence generation {16a}

Eligible patients (i.e., signs of adjustment problems as indicated by the screening questionnaires) will be randomized to the intervention or control group on a 1:1 allocation ratio using computer-generated random numbers. To reduce predictability of a random sequence, a blocking procedure will be used including random block sizes of 4 and 6. In addition, randomization will be stratified by sex, dialysis type, and study site.

### Concealment mechanism {16b}

Randomization will be performed by an independent data manager who is not involved in the study.

The document describing the allocation sequence is not accessible for researchers involved in the study. Only the data manager has access to this document.

### Implementation {16c}

The data manager will generate the allocation sequence. The data manager will inform the researcher of the allocated group to which the participant is randomized. Subsequently, the researcher will inform the participant by telephone and will explain the next steps of the study.

## Assignment of interventions: blinding

### Who will be blinded {17a}

This is an open-label study. Due to the nature of the intervention (ICBT) and the control condition (care as usual), blinding is not required or possible.

### Procedure for unblinding if needed {17b}

Blinding will not occur (see previous point).

## Data collection and management

### Plans for assessment and collection of outcomes {18a}

Participants complete a set of online questionnaires at home. The first assessment is at baseline, the second at 6-month follow-up (i.e., post-treatment for the intervention group) being the primary outcome measure for the analyses, and the third and last assessment is at 12-month follow-up (i.e., 6 months post-treatment for the intervention group). See Table 1 for an overview of the specific questionnaires for each assessment and Fig. 1 for the participant timeline.

### Plans to promote participant retention and complete follow-up {18b}

The assessments consist of online questionnaires that participants can complete at home, at a moment that suits them. To split up completion time, participants can pause the questionnaires at any moment and continue completion within the next 14 days. To promote participant retention in the intervention condition, we will tailor the treatment to the participant’s wishes, needs, and abilities. In addition, the e-Coaches will regularly ask patients for feedback to make adjustments if necessary. This could mean, for example, that for a given patient treatment will include less psychoeducational texts, more practical exercises, and additional phone calls with the e-Coach therapist.

### Data management {19}

Participant identification codes will be used to link data to participants. The file containing the linking between participant numbers and personal data (e.g., name, date of birth) will be managed by the researchers and will be locked for access by others. All the information collected in this trial will be stored in a secured locker. Electronic data will be stored on the central server of the Health, Medical, and Neuropsychology unit of Leiden University, which will be backed up daily. Collected data (personal data from informed consents and medical files, self-report data from questionnaires) will be stored for a period of 15 years.

### Confidentiality {27}

Participant identification codes will be used to link data to participants. The file containing the linking between participant numbers and personal data (e.g., name, date of birth) will be managed by the researchers and will be locked for access by others.

### Plans for collection, laboratory evaluation, and storage of biological specimens for genetic or molecular analysis in this trial/future use {33}

Not applicable, this trial does not have biological specimens.

## Statistical methods

### Statistical methods for primary and secondary outcomes {20a}

Descriptive statistics (means, standard deviations, medians, and interquartile ranges) of relevant variables will be calculated. In case of non-normally distributed variables, transformations will be applied to allow parametric statistics. Baseline data of the intervention and control condition will be compared to assess similarity. Additionally, several *t*-tests will be performed on the 6-month follow-up data, both on the primary and secondary outcomes.

To examine differences in the primary outcome measure change in distress between patients in the intervention and control condition, a linear mixed-model analysis of variance will be conducted. Linear mixed-effects modeling has superior qualities with regard to missing values and makes use of all available data, making this a full-intention-to-treat analysis. Models will be fitted with full information maximum likelihood estimation. Between-group effects at post-treatment and follow-up will be analyzed with baseline scores of dependent variables as covariates. Time will be operationalized as a continuous variable, because post-treatment assessment will vary across participants as a result of different intervention length. Fixed linear effects of time and condition will be included and random effects of intercept.

Similar mixed-model analyses of variance will be conducted to examine differences in change in the scores on the secondary outcome measurements between the two conditions. In addition, correlational and regression analyses will be performed to assess the potential role of demographic and clinical characteristics and psychological parameters (e.g., treatment expectancies, therapeutic relationship) on the effects of the ICBT intervention.

### Interim analyses {21b}

As part of master’s degree education offered at Leiden University, students write their thesis within the context of ongoing research projects and are, for example, involved in the data collection of these projects. We want to offer students the possibility of doing an analysis on the baseline data in a small subsample of participants.

### Methods for additional analyses (e.g., subgroup analyses) {20b}

There are no subgroup analyses planned.

### Methods in analysis to handle protocol non-adherence and any statistical methods to handle missing data {20c}

The primary outcome will be assessed using an intention-to-treat analysis. In addition, we will repeat the analyses with a per protocol analysis using a sample of patients who fully completed the intervention.

### Plans to give access to the full protocol, participant-level data, and statistical code {31c}

The pseudonymized dataset and syntaxes can be made available by the corresponding author upon reasonable request and in accordance with the transfer guidelines of Leiden University.

## Oversight and monitoring

### Composition of the coordinating center and trial steering committee {5d}

The study is centrally organized and coordinated by JT, AWME, and HvM from Leiden University. The principal investigators of the participating centers (HWvH, AG, MMHH, YRPdW, RW, AAMJH) are responsible for the coordination of the study within their centers.

### Composition of the data monitoring committee, its role and reporting structure {21a}

Monitoring of the conduct of the study will be performed by an independent data manager who is not involved in the study. The data manager will check whether the data that have been entered in the database match the source data by random sampling and guarantee the anonymity of the personal data. As per the protocol that has been set up for data management in the Health, Medical and Neuropsychology unit of Leiden University, the data manager will be responsible for checking the data during the recruitment period as well as the final check of the data at the end of the study.

### Adverse event reporting and harms {22}

All adverse events (AEs) reported spontaneously by the participant or observed by the investigator or staff will be recorded. Serious adverse events (SAEs) will be reported by the study staff to the accredited METC that approved the protocol, within 7 days of first knowledge for SAEs that result in death or are life threatening followed by a period of maximum 8 days to complete the initial preliminary report. All other SAEs will be reported within a period of maximum 15 days after the sponsor has first knowledge of the serious adverse events. Reporting suspected unexpected serious adverse reactions (SUSARs) is not applicable, since this study does not include a medicinal product.

### Frequency and plans for auditing trial conduct {23}

The study staff will submit a summary of the progress of the trial to the accredited METC once a year. Information will be provided on the date of inclusion of the first subject, numbers of subjects included, and numbers of subjects that have completed the trial, serious adverse events/ serious adverse reactions, other problems, and amendments.

### Plans for communicating important protocol amendments to relevant parties (e.g., trial participants, ethical committees) {25}

All protocol amendments will be approved of by the METC prior to implementation. If relevant, participants will be informed of protocol modifications.

### Dissemination plans {31a}

Results will be reported in national and international conferences and submitted for publication in peer-reviewed journals.

## Discussion

ICBT is increasingly used in patients with chronic somatic conditions to treat psychological problems such as anxiety and depression and to support patients in adjustment to illness, treatment adherence, and self-management. Results from meta-analyses and systematic reviews suggest promising effects for both psychosocial and disease-specific outcomes of ICBT in several chronic somatic conditions, especially when guided and tailored to specific patient needs [37–39]. However, when it comes to the dialysis population, almost no adequately powered studies and no tailored studies are available.

The few studies that have focused on dialysis patients found improvements in anxiety, depression, and HRQOL, but found mixed results on the experienced burden of kidney disease on daily life and no effects on disability [42, 43]. In terms of the feasibility of the ICBT interventions, challenges were found with regard to computer literacy. Nonetheless, positive results were found with regard to the acceptability of the treatment [42, 44]. Since these results were derived from underpowered and/or non-controlled studies, more research is needed to evaluate the effectiveness of ICBT in dialysis patients.

The current study will assess the effectiveness of a guided ICBT intervention that is specifically developed for dialysis patients and that will be tailored to individual patients’ needs using a multicenter randomized controlled trial. To evaluate the effectiveness of the intervention, we will primarily look at distress, but furthermore take the personalized character of the treatment into account by examining effects by means of a personalized outcome instrument and several secondary HRQOL variables. In addition, the potential role of demographic, clinical, and psychological parameters in determining treatment outcome will be assessed, as well as the cost-effectiveness of the intervention. When the ICBT intervention proves to be effective, the intervention could be implemented in standard care and patients could be offered a personalized treatment supporting them in the problems they prioritize for improvement.

A unique feature of this study is the use of an innovative screening tool to detect patients with an increased risk for adjustment problems such as physical problems, limitations in daily life, and distress. The domains included in the screening tool were based on a previous study of our group in which we evaluated patients’ most prominent problems from a patient perspective [15], to ensure that the screening tool detects what matters most to patients. In addition, the screening tool includes validated screening questionnaires and makes use of disease-specific norm scores, allowing clinicians and patients to compare the individual patient’s scores to other dialysis patients. In previous research, dialysis patients have indicated that they feel reference scores would help to better understand the meaning of their results and indicated to find it highly valuable to receive feedback on their results [9]. To meet these needs, patients’ results will be visualized in a Personal Profile Chart. This chart presents feedback on patients’ results in a way that is easy to understand by using traffic light colors and text boxes with additional explanations [67]. By sending these to all patients who filled out the screening questionnaires, irrespective of whether or not they were eligible for the intervention, patients are provided with feedback on their functioning and are encouraged to discuss this Profile Chart with their nephrologist during a regular consultation.

Another strength of the intervention are the multiple options for tailoring. The ICBT intervention includes modules focusing on issues that patients have indicated to be a priority for improvement as found by a previous study [15], making this intervention a suitable and personally meaningful option for every dialysis patient. Additionally, the intervention will be guided by a therapist, as research showed that therapist-guided interventions yield better results and allows for tailoring of the intervention [19, 40, 75]. At the start of the intervention, the e-Coach uses the patient’s Personal Profile Chart to determine treatment goals together with the patient and, subsequently, tailors the intervention to the patient’s needs. During treatment, the e-Coach continues to tailor the intervention by monitoring whether the form and intensity of the intervention still matches the patient’s preferences and abilities and to make adjustments if necessary.

There are several limitations to consider. As found in previous studies on ICBT in dialysis patients, challenges with computer literacy are likely to occur. This could result in lower inclusion rates due to patients not having access to a computer or Internet. In addition, when patients are enrolled in the study, poor computer skills could lead to drop-out when participating in the study appears to be too difficult. To minimize the risk of drop-out, the therapists who guide the intervention will be highly attentive of these difficulties and will make adjustments if the intervention appears to be too difficult. For example, therapists could choose to communicate via telephone instead of online text messages. Another risk for drop-out is patients’ high disease burden. This risk could be minimized in the same way: regularly monitoring whether ICBT treatment matches the patient’s abilities and making adjustments if necessary (e.g., lowering the intensity).

In conclusion, the present study design will provide insight in the effectiveness of a patient-priorities tailored ICBT intervention in patients with kidney failure who are treated with dialysis. When tailored ICBT proves to be a valuable and effective intervention, the screening procedure and the subsequent ICBT intervention could be implemented in routine care to detect, support, and treat patients struggling with adjustment problems.

## Trial status

Recruitment started in February 2019 and will be ongoing till October 2021. The current protocol is version 7 of 29-1-2020.

## Data Availability

The dataset used and analyzed in this trial will be made available from the corresponding author upon reasonable request.

## Abbreviations

AEs: Adverse events
BMI: Body mass index
CBT: Cognitive-behavioral therapy
CIS: Checklist Individual Strength
CKD: Chronic kidney disease
DSI: Dialysis Symptom Index
DSM: Diagnostic and Statistical Manual of Mental Disorders
E-HELD: E-HEealth treatment in Long-term Dialysis
EPQ-RSS: Eysenck Personality Questionnaire – Revised Short Scale
EQ-5D-5L: Five-level EQ-5D
GAD-7: Generalized Anxiety Disorder 7-item Scale
GFR: Glomerular filtration rate
GP: General practitioner
HRQOL: Health-related quality of life
ICBT: Internet-based cognitive-behavioral therapy
ICECAP-A: ICEpop CAPability measure for Adults
ICQ: Illness Cognition Questionnaire
ISDL: Impact of Chronic Skin Disease on Daily Life
iMTA: Institute for Medical Technology Assessment
iMCQ: iMTA Medical Consumption Questionnaire
iPCQ: iMTA Productivity Cost Questionnaire
ISR: Inventory for Social Resilience
ITRQ: Internet-specific Therapeutic Relationship Questionnaire
LOT-R: Life Orientation Test – Revised
MCS: Mental component score
METC: Medical Research Ethics Committee
METC-LDD: Medical Ethical Committee Leiden-Den Haag-Delft
PCS: Physical component score
PiH: Partners in Health Scale
PHQ-9: Patient Health Questionnaire depression scale
PHQ-ADS: Patient Health Questionnaire Anxiety and Depression Scale
PPPQ: Personalized Priority and Progress Questionnaire
QALYs: Quality-adjusted life-years
QOL: Quality of life
RAND SF -36: RAND Short Form-36 Health Status Inventory
RCT: Randomized controlled trial
SAE: Serious adverse events
SEMCD-6: Self-Efficacy for Managing Chronic Disease 6-ltem Scale
SFQ: Shortened Fatigue Questionnaire
SUSARs: Suspected unexpected serious adverse reactions
VAS: Visual analog scale
